# Remarkable improvement of articular pain by biologics in a Multicentric carpotarsal osteolysis patient with a mutation of *MAFB* gene.

**DOI:** 10.1186/1546-0096-13-S1-P152

**Published:** 2015-09-28

**Authors:** R Nishikomori, T Kawai, K Toshiyuki, H Oda, T Yasumi, K Izawa, O Ohara, T Heike

**Affiliations:** 1 Kyoto University Graduate School of Medicine, Department of Pediatrics, Kyoto, Japan; 2 Aichi Medical University, Department of Pediatrics, Nagakute, Japan; 3 Kazusa DNA Research Institute, Department of Human Genome Research, Chiba, Japan

## Introduction

Multicentric carpotarsal osteolysis syndrome (MCTO) is a rare autosomal dominant disorder, characterized by aggressive osteolysis of the carpal and tarsal bone, and progressive nephropathy leading to end-stage renal disease. Recently, heterozygous mutations in *MAFB* gene within a short region of the amino-terminal transcriptional activation domain had been reported to cause MCTO. Although affected patients suffer from early childhood with a clinical appearance mimicking juvenile idiopathic arthritis, most of anti-rheumatic agents are ineffective to control their pain.

## Patients and methods

A fifteen year old boy was born to healthy parents. There was no family history of consanguinity, skeletal disorders, and rheumatic disorders. He was well until 26 months of age, when he showed claudication symptoms. He was referred for painful and swollen feet, wrists and pes cavus. He also had craniofacial abnormalities of micrognathia, hypotelorism, chubby cheeks and flat face. He showed gradual progression of osteolysis predominantly in the carpal and tarsal bones, and progressive nephropathy with hematoproteinuria. Whole exome sequencing analysis detected a de novo heterozygous mutation in *MAFB* gene which was confirmed by Sanger sequencing. Because this mutation had been reported as a responsible mutation of MCTO, we diagnosed the patient as MCTO caused by the mutation. Until this point, he was treated as a relative disease of juvenile idiopathic arthritis with non-steroidal anti-inflammatory agents, methotrexate, which was not effective to relieve not only osteolysis but articular pain of the patient. At the age of five, he was started to treat with intravenous infliximab by which pain decreased and eventually disappeared in a mean time. Proggressive osteolysis of the carpal and tarsal bone continued and the deformity of fingers was evolved. Therefore, at the age of eight, his biologics was changed to intravenous tocilizumab every 6 weeks. After 3 months treatment, the pain and tenderness in his wrists and fingers disappeared.

## Results

Intravenous tocilizumab every 6 weeks, resulted in remarkable improvement of his articular pain, although osteolysis of the patient remained progressing. The patient became free to pain and could do personal care without difficulty.

## Conclusion

Tocilizumab could be an effective therapy for relief of articular pain of MCTO.

## Consent to publish

Written informated consent for publication of their clinical details was obtained from the patient/parent/guardian/relative of the patient.

